# Onboard experiment investigating metal leaching of fresh hydrothermal sulfide cores into seawater

**DOI:** 10.1186/s12932-018-0060-9

**Published:** 2018-12-06

**Authors:** Shigeshi Fuchida, Jun-ichiro Ishibashi, Kazuhiko Shimada, Tatsuo Nozaki, Hidenori Kumagai, Masanobu Kawachi, Yoshitaka Matsushita, Hiroshi Koshikawa

**Affiliations:** 1 Marine Environment Section, Center for Regional Environmental Research, National Institute for Environmental Studies (NIES), 16-2 Onogawa, Tsukuba, Ibaraki 305-8506 Japan; 2 Department of Earth and Planetary Sciences, Faculty of Sciences, Kyushu University, 744 Motooka, Nishi-ku, Fukuoka 819-0395 Japan; 3 Research and Development (R&D) Center for Submarine Resources, Japan Agency for Marine-Earth Science and Technology (JAMSTEC), 2-15 Natsushima-cho, Yokosuka, Kanagawa 237-0061 Japan; 4 Frontier Research Center for Energy and Resources, The University of Tokyo, 7-3-1 Hongo, Bunkyo-ku, Tokyo, 113-8656 Japan; 5 Department of Planetology, Kobe University, 1-1 Rokkodai-cho, Nada-ku, Kobe, Hyōgo 657-8501 Japan; 6 Ocean Resources Research Center for Next Generation, Chiba Institute of Technology, 2-17-1 Tsudanuma, Narashino, Chiba 275-0016 Japan; 7 Biodiversity Resource Conservation Office, Center for Environmental Biology and Ecosystem Studies, National Institute for Environmental Studies (NIES), 16-2 Onogawa, Tsukuba, Ibaraki 305-8506 Japan; 8 Research Network and Facility Services Division, The National Institute for Materials Science (NIMS), 1-2-1 Sengen, Tsukuba, Ibaraki 305-0047 Japan

**Keywords:** Onboard leaching experiment, Hydrothermal sulfides, Metal contamination, SMS-mining, Marine environmental impacts assessment

## Abstract

We observed the initial release rate of metals from four fresh (i.e., without long time exposure to the atmosphere) hydrothermal sulfide cores into artificial seawater. The sulfide samples were collected by seafloor drilling from the Okinawa Trough by *D/V Chikyu*, powdered under inert gas, and immediately subjected to onboard metal-leaching experiments at different temperatures (5 °C and 20 °C), and under different redox conditions (oxic and anoxic), for 1–30 h. Zinc and Pb were preferentially released from sulfide samples containing various metals (i.e., Mn, Fe, Cu, Zn, Cd, and Pb) into seawater. Under oxic experimental conditions, Zn and Pb dissolution rates from two sulfide samples composed mainly of iron disulfide minerals (pyrite and marcasite) were higher than those from two other sulfide samples with abundant sphalerite, galena, and/or silicate minerals. Scanning electron microscopy confirmed that the high metal-releasing sample contained several galvanic couples of iron disulfide with other sulfide minerals, whereas the low metal-releasing sample contained fewer galvanic couples or were coated by a silicate mineral. The experiments overall confirmed that the galvanic effects with iron disulfide minerals greatly induce the initial release of Zn and Pb from hydrothermal sulfides into seawater, especially under warm oxic conditions.

**Electronic supplementary material:**

The online version of this article (10.1186/s12932-018-0060-9) contains supplementary material, which is available to authorized users.

## Introduction

Sulfide minerals, which are economically important metal resources, have become a major source of contamination via the release of metal cations and acids by oxidation [1]. There has recently been a focus on seafloor massive sulfide (SMS) deposits associated with hydrothermal venting as new mining targets. However, anthropogenic release of sulfide minerals into marine environments associated with SMS-mining operations may result in the generation of metal and acid contaminated seawater [2–5].

The mechanisms and kinetics of the oxidation of individual sulfide minerals in aqueous media, particularly pyrite (FeS_2_) [6, 7], sphalerite (ZnS) [8–10], and galena (PbS) [11], have been extensively investigated in the context of terrestrial mining. The results show that the oxidation rates of these minerals change depending on the physicochemical parameters; namely, pH, temperature, redox potential, oxygen pressure, and amounts of other oxidizing agents such as ferric ions. Based on these findings, the oxidation of terrestrial sulfides can be significantly promoted by atmospheric oxygen. However, the release of metals from hydrothermal sulfides into seawater is considered to be limited in open marine environments because of the alkalescence, high-buffering capacity, and low gaseous oxygen content of seawater [5].

Several studies have recently investigated potential leaching of metals and metalloids into seawater from natural hydrothermal sulfides (i.e., complex mixtures of various sulfide minerals) collected around active/inactive vent chimneys. Simpson et al. [12] investigated metal leaching from hydrothermal sulfides of both active and inactive vent chimneys in the East Manus Basin hydrothermal field (Papua New Guinea) as part of the Solwara 1 project. In their study, they reacted large chips of sulfide samples (25 mm diameter) with seawater at 22 °C to give a liquid to solid ratio of 1:10, resulting in the rapid release of Zn (78–430 µM), Pb (0.60–1.6 µM) and Cu (< 0.10–0.90 µM) from those samples into seawater within 12 min. Parry [13] observed that a Fe-rich hydrothermal sulfide sample collected from the East Manus Basin field released a high amount of Zn into seawater (150 µM) along with small amounts of Cu, Cd, and Pb (< 10 µM) during leaching for 3 h at 24 °C to give a liquid to solid ratio of 1:10. Fuchida et al. [4] conducted leaching experiments using four hydrothermal sulfide samples (i.e., Fe–Zn–Pb-rich, Ba-rich, Fe-rich, Zn–Pb-rich samples) collected from the Okinawa Trough hydrothermal fields (Japan). Specifically, they used fine particulate matters (< 1/16 mm) of those sulfide samples, which would contribute to plume formation during SMS-mining operation. When these particulate samples were reacted with seawater at 25 °C at a liquid to solid ratio of 1:20, Zn (870–70,000 µM), Pb (< 220 µM) and Cu (< 480 µM) were rapidly released into oxic seawater within five min, and these levels were significantly higher than those from the large chip samples used by Simpson et al. and Parry [12, 13].

These results imply that natural hydrothermal sulfides could be a potential source of metal contaminants in marine environments that is likely to release metals at higher levels than the water quality chronic criterion for marine organisms (1.2 µM for Zn, 0.039 µM for Pb and 0.049 µM for Cu) proposed by the United States Environmental Protection Agency [14]. However, the hydrothermal sulfide surfaces used in previous studies were exposed to atmospheric oxygen during long-term storage of samples before the leaching experiments; thus, sulfide minerals would have been oxidized and transformed into more labile states [4] and may have increased the release of metals in seawater. Therefore, the interactions of purely fresh hydrothermal sulfides before long-term exposure to atmospheric oxygen with seawater need to be investigated to enable adequate and realistic evaluation of the leaching potential of natural hydrothermal sulfides in marine environments.

In this study, fresh hydrothermal sulfides were collected from the Izena Hole, Okinawa Trough by *D/V Chikyu* and immediately subjected to leaching experiments onboard. The data were then used to evaluate the potential for metal leaching from hydrothermal sulfide into seawater before long-term exposure to atmospheric oxygen. Ground sulfide samples were used for the leaching experiment because fine suspended sulfide is considered to have the ability to release large amounts of metals via interaction of their high specific surface area with seawater [3]. Furthermore, different temperatures (5 °C and 20 °C) and redox conditions (oxic and anoxic) were used to evaluate the release of metals at both the surface (20 °C and oxic) and seafloor (5 °C and anoxic). Based on the experimental results, we assessed the potential generation of metal rich seawater by SMS-mining operations.

## Materials and methods

### Sample preparation of hydrothermal sulfides

**Fig. 1:**
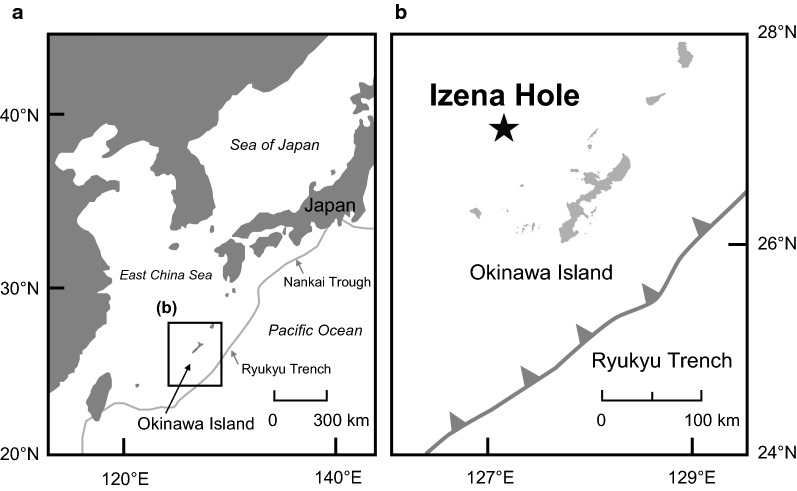
Maps of **a** of Okinawa Island in Japan with the Nankai Trough and Ryukyu Trench systems and **b** drilled sites at the Izena Hole by *D/V Chikyu* during Expedition 909 (CK16-05 Cruise) (**b** was modified after Fuchida et al. [4])

The hydrothermal sulfide samples used for leaching experiments were obtained by seafloor drillings at the Izena Hole (27°14′N 127°04′E) in the middle Okinawa Trough by the *D/V Chikyu* during Expedition 909 (CK16-05 Cruise, 16 Nov–15 Dec, 2016) (Fig. 1). Sediment cores were collected from three drilled holes (Holes C9026A, C9027B, and C9028A). Hole C9027B is located on a central part of the active hydrothermal mound. Holes C9026A and C9028A are at the flank of the mound, about 80 and 60 m east, respectively, of Hole C9027B. Four samples (C9026A 7X-CC, C9027B 1X-CC, C9028A 7S-CC, and C9028A 1H-7) used for leaching experiments were taken from sections rich in sulfide minerals. Photographs of those samples are shown in Additional file 1: Figure S1. C9027B 1X-CC [0.14–0.17 m below the seafloor (mbsf)] (Additional file 1: Figure S1b) and C9028A 7S-CC (6.80–6.85 mbsf) (Additional file 1: Figure S1c) were the upper sections of the drilled holes, while C9026A 7X-CC (35.71–35.78 mbsf) (Additional file 1: Figure S1a) and C9028A 1H-7 (41.17–41.22 mbsf) (Additional file 1: Figure S1d) were the lower sections of the drilled holes. The surfaces of the sulfide samples were quickly rinsed with Milli-Q water to remove drilling mud, after which excess moisture was wiped off. Each sample was then manually crushed with a tungsten carbide mortar and pestle, then ground with an agate mortar and pestle under a N_2_ atmosphere to avoid sample oxidation. The ground samples of C9026A 7X-CC, C9027B 1X-CC, C9028A 7S-CC, and C9028A 1H-7 were denoted CKL-1, CKL-2, CKL-3, and CKL-4, respectively, as shown in Table 1. Although these ground samples were slightly moist, we did not dry them to prevent alteration of the original constituents of mineral assemblages to other secondary minerals.

**Table 1:** Sample codes and physical properties of powdered samples used in leaching experiments

Sample ID	Hole, core section	Depth (mbsf*)	Grain size range, mode (µm)	Surface area (m^2^ g^−1^)
CKL-1	C9026A, 7X-CC	35.71–35.78	0.10–290, 21	0.45
CKL-2	C9027B, 1X-CC	0.14–0.17	1.6–230, 17	0.41
CKL-3	C9028A, 7S-CC	41.17–41.22	1.9–190, 14	1.0
CKL-4	C9028A, 1H-7	6.80–6.85	1.6–67, 14	0.71

*mbsf meters below seafloor

### Physical properties of ground sulfide particulates

The grain size and Brunauer–Emmett–Teller (BET) surface area (*A*_BET_) of the ground sulfide samples were measured using a laser diffraction particle size analyzer (SALD-2100, Shimadzu, Co., Kyoto, Japan) and a high-precision gas/vapor adsorption measurement instrument (BELSORP-max, MicrotracBEL Corp., Osaka, Japan), respectively, at the National Institute for Materials Science (NIMS; Tsukuba, Japan). The mode values of the grain sizes for samples CKL-1, CKL-2, CKL-3, and CKL-4 were 21, 17, 14, and 14 µm, respectively, while the respective surface areas were 0.45, 0.41, 1.0, and 0.71 m^2^ g^−1^ (Table 1).

### Chemical compositions and mineralogy of sulfide samples

The chemical compositions of the samples were determined by inductively coupled plasma-mass spectrometry (ICP-MS; 8800 ICP-QQQ, Agilent Technologies, Inc., Santa Clara, CA, USA) after digestion with HCl/HClO_4_/HF/HNO_3_ according to the method reported by Fuchida et al. [4] (Table 2a). The mineral assemblages of the samples were determined by X-ray diffraction (XRD; MiniFlex600, Rigaku, Tokyo, Japan, at NIMS) with Ni-filtered monochromatic Cu Kα radiation at 2*θ* angles between 5° and 80° (Table 2b).

**Table 2:** (a) Chemical compositions and (b) mineral assemblages of hydrothermal sulfide samples

(a)						
Sample ID	Concentration (mmol kg^−1^)					
	Mn	Fe	Cu	Zn	Cd	Pb
CKL-1	3.5	2500	96	4600	11	970
CKL-2	5.8	5800	56	1700	3.2	370
CKL-3	1.9	7600	100	370	0.43	12
CKL-4	7.9	5700	56	3200	8.6	170

(b)								
Sample ID	Pyrite	Galena	Sphalerite	Marcasite	Barite	Magnesite	K-feldspar	Wollastonite
CKL-1	+	+++	+++				+	+
CKL-2	+++	++	++			+		
CKL-3	++++				+			
CKL-4	++	+	+++	+++				

++++: dominant, +++: abundant, ++: common, +: rare

The CKL-1 contained 4600 mmol kg^−1^ Zn, 2500 mmol kg^−1^ Fe, 970 mmol kg^−1^ Pb, and 96 mmol kg^−1^ Cu, and consisted mainly of sphalerite and galena, with little amounts of pyrite, K-feldspar, and wollastonite. The CKL-2 contained 5800 mmol kg^−1^ Fe, 1700 mmol kg^−1^ Zn, 370 mmol kg^−1^ Pb, and 56 mmol kg^−1^ Cu, and mainly consisted of pyrite, with small amounts of sphalerite and galena. The CKL-3 contained 7600 mmol kg^−1^ Fe, 370 mmol kg^−1^ Zn, and 100 mmol kg^−1^ Cu, and pyrite was the predominant mineral. The CKL-3 had the lowest Pb content (12 mmol kg^−1^) among all samples. The CKL-4 contained 5700 mmol kg^−1^ Fe, 3200 mmol kg^−1^ Zn, 170 mmol kg^−1^ Pb, and 56 mmol kg^−1^ Cu, and consisted mainly of sphalerite, marcasite, and pyrite.

### Onboard leaching experiments

**Fig. 2:**
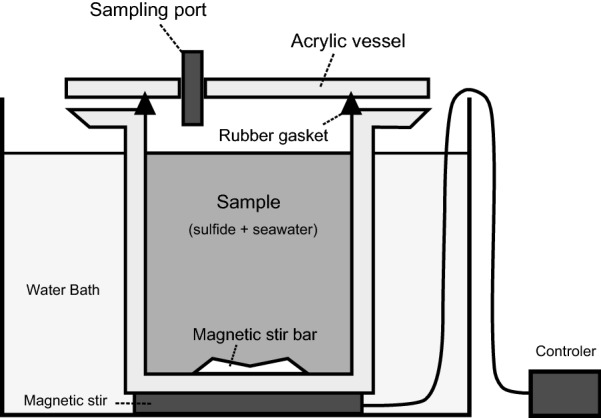
Schematic of the experimental set up. The ground sulfide sample (3 g) was placed with the artificial seawater (150 mL) in the cylindrical acrylic vessel (250 mL), and then the vessel was tightly closed with atmospheric air or N_2_ gas. The mixture was stirred with the PTFE magnetic stirring bar, and the temperature was kept at 5 °C or 20 °C in the water bath. The samples solution was collected with disposable syringe from the sampling port. Sampling from anoxic systems was conducted inside the N_2_ chamber

The four ground sulfide samples were reacted under four different sets of conditions (anoxic at 5 °C, anoxic at 20 °C, oxic at 5 °C, and oxic at 20 °C). Each ground sample (3 g) was mixed with artificial seawater (150 mL) containing 3.2% NaCl, 0.35% MgSO_4_, and 0.017% NaHCO_3_ (pH = 8.1) in a cylindrical acrylic vessel (250 mL). The schematic of the experimental set-up is shown in Fig. 2. For oxic experiments (i.e., high redox conditions), the vessel was closed with atmospheric air. For the anoxic experiments (i.e., low redox conditions), the artificial seawater was degassed in a vacuum before mixing with ground sulfide samples, after which the vessel was filled with N_2_ gas. All systems were prepared in duplicate to check the reproducibility of the experiments. The mixture was stirred with a poly(tetrafluoroethylene) (PTFE) magnetic stirring bar and the temperature was kept at 5 °C or 20 °C in a water bath. A small portion of the solution (10 mL) was removed at 1, 4, 10, 18, and 30 h with a disposable syringe. Sampling from anoxic systems was conducted inside the N_2_ chamber. The images of experimental operations are shown in Additional file 1: Figure S2. An aliquot of the samples (5 mL) was filtered through a PTFE membrane filter (0.45 µm) and preserved with HNO_3_ (1%) in a polypropylene tube for subsequent determination of the metal composition by ICP-MS analysis (see next section). The rest of the sample was used for pH and ORP measurements. The pH values of seawater were measured using a pH meter (Horiba D-75, Horiba, Ltd., Kyoto, Japan) that had been calibrated on the National Bureau of Standards (NBS) scale at each experimental temperature using two standard buffer solutions, phthalate and phosphate equimolar solutions (Wako Pure Chemical Industries, Ltd.). The oxidation-reduction potential (ORP), i.e., *E*_h_ value, of seawater was measured using an ORP electrode with an KCl-Ag/AgCl system (Horiba 9300-10D, Horiba, Ltd., Kyoto, Japan), and the measured value was corrected to the hydrogen potential (i.e., *E*_h_ vs. standard hydrogen electrode, SHE). All sample and reagent bottles and reaction vessels were cleaned with 2 N HNO_3_ before use.

### Analysis of dissolved metals in seawater

Before ICP-MS analysis, the sample solution was desalinated with a chelating resin packed in a polypropylene syringe column (7 mL) (Nobias Chelate PA1, Hitachi-High Tech Fielding Corporation, Tokyo, Japan) [15]. The resin had both iminodiacetic and ethylenediaminetriacetic acid moieties, which can selectively capture transition metals (excluding alkali and alkaline-earth metals), metalloids except for aluminum, and halogens. Before injection of sample solution to the column, the packed resin was pre-cleaned with acetone, 3 N HNO_3_, and ultrapure water, then conditioned with 0.1 M CH_3_COONH_4_ buffer solution (pH 5.5). The pH of the sample solution was adjusted to 5.5 with NH_4_OH, after which the solution was injected into the cleaned resin column. The resin was subsequently rinsed with ultrapure water, after which the captured metal fraction was eluted with 3 N HNO_3_ (3 mL). The obtained solution was then diluted ten-fold with ultrapure water, after which dissolved metals present at detectable levels in the obtained solution were identified by ICP-MS. Among the capturable metals, Fe, Cu, Zn, and Pb were quantifiable by ICP-MS. A value ten times the standard deviation of the matrix control blank was defined as the limit of quantification (nM) (2.7 for Fe, 1.7 for Cu, 0.11 for Zn, and 0.25 for Pb).

To determine the recoveries of metals during desalination treatment, four metal standards (Fe, Cu, Zn, and Pb) were added to the artificial seawater used in the leaching experiment. These solutions were flowed through a chelating resin in the same manner, after which the recoveries were determined based on the results of ICP-MS analysis. The recoveries of Fe, Cu, Zn, and Pb after three rounds were all > 96%, and the analytical error was within ± 3% during this procedure. Blank artificial seawater was also eluted through the resin in the same manner to evaluate eventual contaminants during desalination. Based on ICP-MS analysis, those metals were below the quantifiable limits.

### Examination of mineral particulates by scanning electron microscopy–energy-dispersive X-ray spectroscopy

Fragments of the sulfide particulates collected from the same core used for the leaching experiment were examined by scanning electron microscopy–energy-dispersive X-ray spectroscopy (SEM–EDS; JSM7001F, JOEL Ltd., Tokyo, Japan) at Kyushu University. Mineral fragments were molded in resin and polished to obtain a smooth surface, after which the mounts were observed by the SEM in secondary electron (SE) and backscattered electron (BSE) modes. The chemistry of the major elements was analyzed using the attached EDS.

## Results

### Changes in *E*_h_ and pH values of seawater

The initial *E*_h_ values of seawater in the oxic and anoxic experiments were 0.33–0.39 and 0.10–0.19 V, respectively (Additional file 1: Figure S3). Over the period of the leaching experiments (1–30 h), these *E*_h_ values varied slightly, but the differences between the oxic and anoxic systems were fairly constant, indicating that the projected redox condition in each system was almost maintained.

**Fig. 3:**
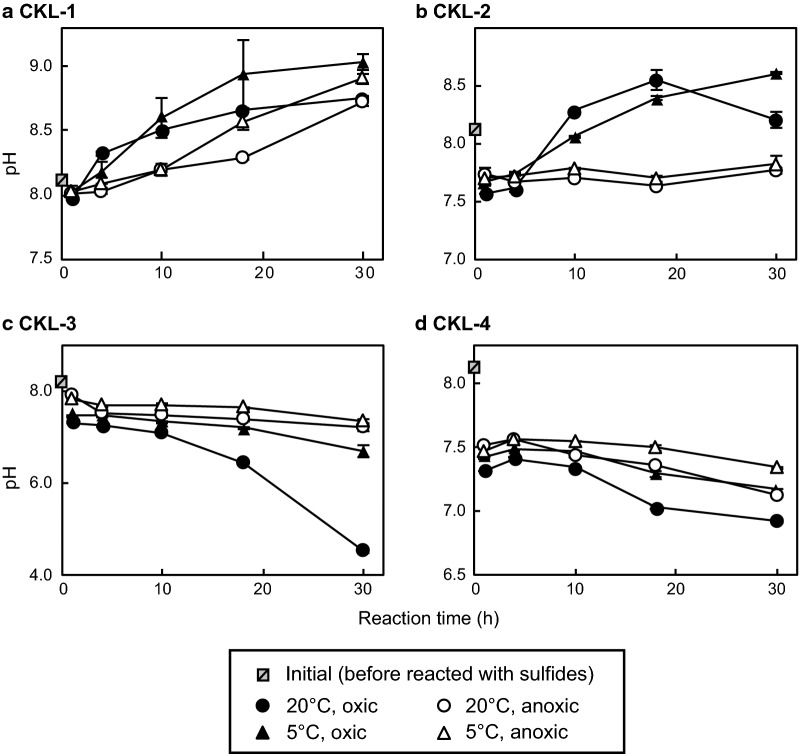
Changes in pH for **a** CKL-1, **b** CKL-2, **c** CKL-3, and **d** CKL-4 under different redox and temperature conditions over time. Plots show mean values of duplicates, and error bars indicate ranges of duplicates (difference between max and min values)

The pH varied differently under each experimental condition. The pH values of the CKL-1 solutions showed a continuous increase from 8.1 to 8.7–9.0 during reaction for 30 h under all experimental conditions (Fig. 3a). For the CKL-2 solution, the pH initially decreased from 8.1 to 7.6–7.7 in the first hour under all experimental conditions, after which it increased gradually to 8.2–8.6 under oxic conditions during 1–30 h, while it remained constant under anoxic conditions (Fig. 3b). For the CKL-3 solution, the pH decreased initially from 8.1 to 7.3–7.9 in the first hour under all experimental conditions, then decreased gradually to 6.7–7.4 under oxic conditions at 5 °C and anoxic conditions at both 5 °C and 20 °C, whereas it decreased greatly to 4.5 under oxic conditions at 20 °C (Fig. 3c). The pH of the CKL-4 solution showed an initial decrease from 8.1 to 7.8–7.4 in the first hour, after which it decreased slightly to 6.9–7.4 under all experimental conditions (Fig. 3d).

### Dissolved metal concentrations in seawater

**Fig. 4:**
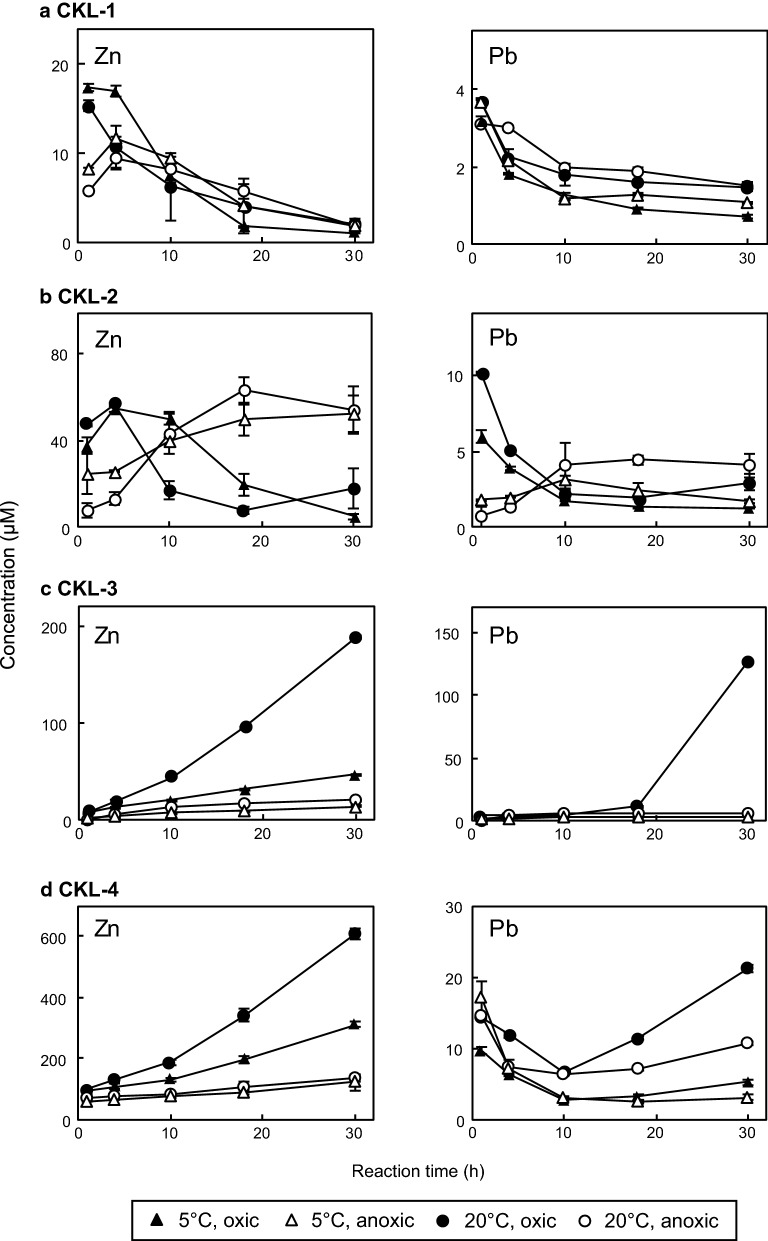
Changes in concentrations of dissolved Zn and Pb for **a** CKL-1, **b** CKL-2, **c** CKL-3, and **d** CKL-4 under different redox and temperature conditions over time. Plots show mean values of duplicates, and error bars indicate ranges of duplicates (difference between max and min values)

Zinc and Pb in the reacted solution were quantifiable under all experimental conditions, and these concentrations differed depending on the reaction temperature and redox conditions (Fig. 4). As concentrations of Cu and Fe in the solution were below the quantifiable limits under most of the experimental conditions (2.7 nM for Fe and 1.7 nM for Cu), we focused on Zn and Pb in this section.

The Zn concentrations in the CKL-1 solution under all experimental conditions (Fig. 4a) were lower (1.1–17 µM) than those of other samples over the experimental periods. For the CKL-2 solution, the Zn concentrations under oxic conditions (38–57 µM) were higher than those under anoxic conditions (7.9–26 µM) for the initial 4 h at both 5 °C and 20 °C (Fig. 4b). The concentrations under oxic conditions then decreased to 5.0–18 µM after 30 h, whereas those under anoxic conditions increased until finally reaching 52–54 µM. For the CKL-3 and CKL-4 solutions, large monotonic increases in the Zn concentrations were observed (Fig. 4c, d). The final Zn concentrations in both the CKL-3 and CKL-4 solutions were highest under oxic conditions at 20 °C (190–610 µM), followed by oxic conditions at 5 °C (47–310 µM), anoxic conditions at 20 °C (21–140 µM), and anoxic conditions at 5 °C (15–120 µM).

The Pb concentrations in the CKL-1 solution were lower than the Zn concentrations, ranging from 0.74 to 3.7 µM under all conditions (Fig. 4a). For the CKL-2 solution, the Pb concentrations under oxic conditions (3.9–10 µM) were higher than those under anoxic conditions (0.76–2.0 µM) during the initial 4 h at both 5 °C and 20 °C (Fig. 4b). The concentrations under oxic conditions then decreased to 1.3–3.0 µM at 30 h and became lower than those under anoxic conditions (1.8–4.1 µM). For the CKL-3 solution, a large amount of Pb was released under oxic conditions at 20 °C during 18–30 h, while the Pb concentration was 12 µM at 18 h, then increased greatly to 130 µM at 30 h (Fig. 4c). The Pb concentrations in the CKL-4 solution after the first hour were 9.7–17 µM, after which they increased to 21 µM under oxic conditions at 20 °C and then to 3.2–11 µM under oxic conditions at 5 °C and under anoxic conditions at both 5 °C and 20 °C (Fig. 4d).

Although Fe and Cu were not found in the reacted solutions under most of the experimental conditions, high concentrations of Fe (129 µM) and Cu (23 µM) were found in the CKL-3 solution under oxic conditions at 20 °C after 30 h (Additional file 1: Table S1).

### Morphologies of hydrothermal sulfide particulates

**Fig. 5:**
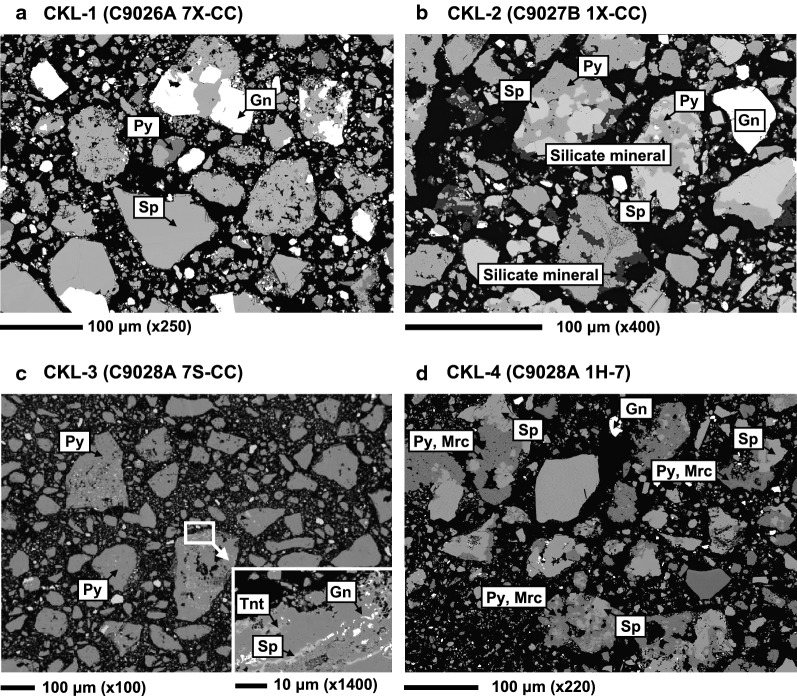
BSE images by SEM–EDS observation of mineral particulates for **a** CKL-1 (C9026A 7X-CC), **b** CKL-2 (C9027B 1X-CC), **c** CKL-3 (C9028A 7S-CC), and **d** CKL-4 (C9028A 1H-7). Mineral names are abbreviated as follows: Py, pyrite; Sp, sphalerite; Gn, galena; Mrc, marcasite; Tnt, tennantite

Figure 5 shows typical sectional BSE images obtained by SEM–EDS analysis of four ground sulfide samples. Sulfide mineral species were identified based on their chemical compositions and mineral assemblages determined by EDS and XRD, respectively. The BSE images revealed that CKL-1 mainly consisted of sphalerite conjugated with galena and a small amount of pyrite (Fig. 5a). The XRD results imply the presence of small amounts of K-feldspar and wollastonite in the CKL-1 particulates (Table 2), but these minerals were not observed by SEM. For CKL-2, sphalerite, pyrite, and a small amount of galena were present adjacent to each other in the mineral particulates (Fig. 5b). In addition, the CKL-2 particulates were often coated by a silicate mineral layer. The CKL-4 consisted of sphalerite, galena, and two iron disulfide minerals, pyrite and marcasite (Fig. 5d). We could not discriminate between the two iron disulfide minerals morphologically. The CKL-3 particulates were mainly pyrite, which agrees with the XRD results. However, we observed small pieces of sphalerite, galena, and tennantite [(Cu, Fe)_12_As_4_S_13_] in the pyrite particulates in the magnified image of the CKL-3 sample (Fig. 5c). Highly soluble Zn- and Pb-sulfate minerals (e.g., anglesite) were not found in any samples.

## Discussion

**Fig. 6:**
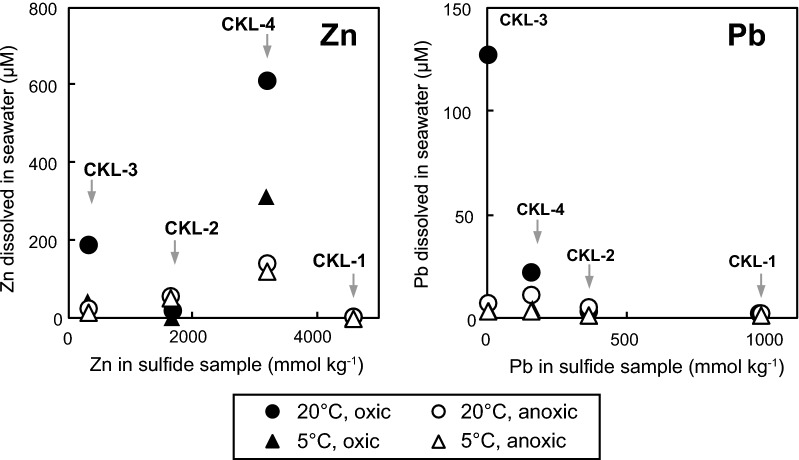
Amounts of Zn and Pb in hydrothermal sulfide samples (mmol kg^−1^) before the experiment and in solutions (µM) reacted for 30 h under different redox and temperature conditions

Our onboard leaching experiments showed that hydrothermal sulfides released Zn and Pb into seawater, especially under oxic conditions at 20 °C, without long term exposure to the atmosphere. X-ray diffraction analysis revealed high contents of sphalerite, galena, and pyrite/marcasite in the hydrothermal sulfide samples, and the mineral assemblages were qualitatively consistent with the chemical compositions of the samples (Table 2). If simple oxidation reactions (MS + 2O_2_ → M^2+^ + SO_4_^2−^, M = divalent metal) of these sulfide minerals were the main reaction to release metals from the sulfide samples into seawater, the metal concentrations in the seawater would have increased with increasing the metal contents in the sulfide samples. However, as shown in Fig. 6, the final concentrations of Zn and Pb in the seawater were rarely correlated with those in the sulfide samples before the experiments (Fe was not detected under most of the experimental conditions). These discrepancies of metal compositions between the sulfide samples before experimentation and after seawater reacted with those samples suggest that additional reactions may would be involved in the dissolution of metals from the hydrothermal sulfides into seawater. In this section, we discuss possible mechanisms for the preferential release of Zn and Pb from hydrothermal sulfides into seawater without long term exposure to the atmosphere.

### Effects of solubility of each metal on its composition in seawater

As described above, dissolved Fe was absent from the reacted seawater except for the case of CKL-3 under oxic conditions at 20 °C after 30 h (Additional file 1: Table S1), even though Fe was present in high concentration in the sulfide samples (2500–7600 mmol kg^−1^). It has been reported that oxidative dissolution of pyrite (FeS_2_ + 7/2O_2_ + H_2_O → Fe^2+^ + 2SO_4_^2−^ + 2H^+^) can proceed even in seawater, with an oxidation rate of 2.0 × 10^−8^–5.0 × 10^−10^ mol kg^−1^ s^−1^ [16]. Sphalerite also often contains large amounts of Fe as impurities [17]; thus, it is likely that ferrous ion (Fe^2+^) was released from hydrothermal sulfides into seawater via oxidation of pyrite and sphalerite during experimentation. However, the ferrous ion was likely instantly oxidized to ferric ion (Fe^3+^), which has low solubility in seawater (only 0.2–0.3 nM) [18] and precipitated into insoluble oxyhydroxides such as FeO(OH). Although we did not quantify the Fe precipitate formed during the reaction, the absence of Fe in the solutions after passing through a 0.45 µm filter indicates that Fe likely precipitated formed during the experiment.

In turn, Zn and Pb are relatively soluble compared to Fe. Indeed, the solubility of Pb is reportedly 3 µM in seawater [19]. Zinc concentrations in seawater are also expected to be high because most of the secondary Zn-minerals are highly soluble [20] (its solubility in seawater has not been reported). Therefore, the increase in the Zn and Pb concentrations in seawater during experimentation was likely due to the higher solubility of these metals in seawater.

### Dissolution rates of Zn and Pb from hydrothermal sulfides into seawater

To determine which reaction pathway mainly regulated the concentrations of Zn and Pb in each system, we calculated the average of the apparent dissolution rate (*R*) of those metals from sphalerite (*R*_ZnS_ mol m^−2^ s^−1^) and galena (*R*_PbS_ mol m^−2^ s^−1^) in the hydrothermal sulfide sample (i.e., mixture of various sulfide minerals) using the equation below; the results are shown in Table 3.

**Table 3:** Dissolution rates of ZnS (*R*_ZnS_) and PbS (*R*_PbS_) in sulfide samples during 1–30 h

Sample ID			*R*_ZnS_ (mol m^−2^ s^−1^)	*R*_PbS_ (mol m^−2^ s^−1^)			*R*_ZnS_ (mol m^−2^ s^−1^)	*R*_PbS_ (mol m^−2^ s^−1^)
CKL-1	Anoxic	5 °C	−3.6 × 10^−11^ (−3.5 to − 3.7 × 10^−11^)	−3.0 × 10^−11^ (−2.9 to − 3.1 × 10^−11^)	Oxic	5 °C	−8.7 × 10^−11^ (−8.6 to − 8.9 × 10^−11^)	−2.8 × 10^−11^ (−2.7 to − 2.9 × 10^−11^)
		20 °C	−2.4 × 10^−11^ (−2.3 to − 2.4 × 10^−11^)	−2.1 × 10^−11^ (−2.0 to − 2.1 × 10^−11^)		20 °C	−7.2 × 10^−11^ (−7.1 to − 7.3 × 10^−11^)	−2.7 × 10^−11^ (−2.6 to − 2.7 × 10^−11^)
CKL-2	Anoxic	5 °C	6.0 × 10^−10^ (4.7 to 7.2 × 10^−10^)	−4.8 × 10^−11^ (−4.1 to − 5.5 × 10^−11^)	Oxic	5 °C	−1.5 × 10^−9^ (−1.4 to − 1.6 × 10^−9^)	−4.0 × 10^−10^ (−3.8 to − 4.3 × 10^−10^)
		20 °C	1.4 × 10^−9^ (1.1 to 1.6 × 10^−9^)	1.8 × 10^−10^ (1.7 to 1.9 × 10^−10^)		20 °C	−1.5 × 10^−9^ (−1.2 to − 1.8 × 10^−9^)	−6.4 × 10^−10^ (−6.2 to − 6.7 × 10^−10^)
CKL-3	Anoxic	5 C	3.4 × 10^−9^ (3.2 to 3.5 × 10^−9^)*	5.7 × 10^−8^ (4.1 to 7.4 × 10^−8^)*	Oxic	5 °C	1.1 × 10^−8^ (1.0 to 1.1 × 10^−8^)*	− 3.1 × 10^−8^ (−2.5 to − 3.7 × 10^−8^)*
		20 °C	8.1 × 10^−9^ (7.9 to 8.4 × 10^−9^)*	2.5 × 10^−7^ (2.3 to 2.8 × 10^−7^)*		20 °C	4.4 × 10^−8^ (4.3 to 4.4 × 10^−8^)*	2.9 × 10^−7^ (2.8 to 2.9 × 10^−7^)*
CKL-4	Anoxic	5 °C	4.9 × 10^−10^ (3.4 to 6.4 × 10^−10^)	−3.3 × 10^−9^ (−2.9 to − 3.8 × 10^−9^)	Oxic	5 °C	1.7 × 10^−9^ (1.6 to 1.7 × 10^−9^)	−1.3 × 10^−9^ (−1.3 to − 1.4 × 10^−9^)
		20 °C	5.6 × 10^−10^ (4.7 to 6.4 × 10^−10^)	−1.4 × 10^−9^ (−1.4 to − 1.6 × 10^−9^)		20 °C	3.9 × 10^−9^ (3.8 to 4.0 × 10^−9^)	2.7 × 10^−10^ (2.6 to 2.9 × 10^−10^)

The *R*_ZnS_ and *R*_PbS_ values with an asterisk* for CLK-3 were calculated during 1–18 h because the pH decreased greatly to 4.5 under oxic conditions at 20 °C during 18–30 h. The values represent means (ranges) of duplicates

1 $${\text{R = }}\frac{\text{a}}{{\left( {A_{BET} \cdot r} \right) \cdot {\text{m}}}} ( {\text{mol m}}^{ - 2} {\text{s}}^{ - 1} )$$

where *a* is the slope (mol l^−1^ s^−1^) of the linear regression of the dissolved Zn and Pb concentrations versus sampling times (1 to 30 h). The *a* value for the CKL-3 was calculated during 1–18 h because the pH decreased greatly from 6.5 to 4.5 under oxic conditions at 20 °C between 18 and 30 h, probably owing to the great difference in chemical conditions compared with the other reaction systems. *A*_BET_ (m^2^ g^−1^) is the specific surface area of each sulfide sample per a unit mass, and *r* (−) is the weight ratio of the mineral of interest (*r*_ZnS_ for sphalerite and *r*_PbS_ for galena) to the sulfide samples. The *r*_ZnS_ and *r*_PbS_ values of each sample were estimated from the Zn and Pb concentrations in the sulfide samples (Table 2a) assuming that all amounts of those metals in the sulfide samples were present as sphalerite and galena, respectively. The product of *A*_BET_ and *r* provides the specific surface area of each mineral in the sulfide samples. *m* (g L^−1^) is the mass of sphalerite and galena in the sulfide samples present in a unit volume of the reaction solution. The normalized *R* allows us to compare the dissolution behavior of sphalerite and galena among different reaction systems. If the assumption for *r*_ZnS_ and *r*_PbS_ values described above is satisfied, our defined *R* should be comparable to that defined in previous studies [10, 11, 21, 22] that evaluated the dissolution rate of individual minerals, but not hydrothermal sulfides (mixture).

The calculated *R*_ZnS_ and *R*_PbS_ of the CKL-1 sample showed negative values under experimental conditions (Table 3). Negative *R*_ZnS_ and *R*_PbS_ values were also observed in CKL-2 under oxic conditions at both 5 °C and 20 °C. These negative values indicate that metal release from sulfide minerals was limited and that removal reactions of initially released metals in seawater became dominant during the reaction of 1–30 h. Such declines in the dissolution rates of sulfide minerals were observed in several previous studies that showed single sulfide mineral dissolutions [10, 11]. The authors explained that the formation of insoluble hydroxides and passive layers on sulfide mineral surfaces could decrease dissolution rates [10, 11]. The concentrations of Pb in the solution that had negative *R*_PbS_ values were 3.1–10 µM during the first hour, which were higher than the soluble concentrations in seawater (approximately 3 µM [19]). Although precipitates were not determined after experimentation, insoluble salts such as PbSO_4_ and PbCO_3_ could be formed [19, 20] in seawater, resulting in negative *R*_PbS_ values for the CKL-1 and CKL-2 solutions. For negative *R*_ZnS_ values, removal reactions other than precipitation are considered to be the primary contributors because most of the secondary Zn-minerals are highly soluble [20]. For example, Zn^2+^ might be removed from seawater by adsorption onto other precipitates such as FeO(OH), which has a high specific surface area [23].

In contrast to CKL-1 and CKL-2, positive *R*_ZnS_ values were observed for CKL-3 and CKL-4 under all experimental conditions, although the *R*_PbS_ values were negative under oxic conditions at 5 °C. These positive values indicate that dissolution of hydrothermal sulfide minerals to seawater are the dominant reaction in CKL-3 and CKL-4. The highest *R*_ZnS_ values were obtained under oxic conditions at 20 °C for both CKL-3 and CKL-4.

The simple oxidation reactions of sphalerite can be represented by the following equations

2 $${\text{ZnS }} + {\text{ 2O}}_{ 2} \to {\text{Zn}}^{ 2+ } + {\text{ SO}}_{ 4}^{ 2- }$$

Although dissolved ferric ions can also greatly accelerate the oxidation of sulfide minerals [10, 11, 24], ferric ion, as described above, has low solubility (only 0.2–0.3 nM) and is rarely present in seawater [18]. Iron was not detected in reacted seawater under most experimental conditions (Additional file 1: Table S1), suggesting that oxidation by ferric ions likely made a minor contribution to the results observed in our experiments.

The simple oxidation rate of sphalerite in ultrapure water with an initial pH of 6 at room temperature (in the absence of ferric ions) was reported to be 2.0 × 10^−10^ mol m^−2^ s^−1^ [10] based on the SO_4_^2−^ concentration in the solution. The oxidation rates of individual sulfide minerals generally decrease with increasing pH [3, 5, 25, 26]; thus, the oxidation rates of sphalerite in alkalescent seawater should be lower than the reference value. However, the calculated *R*_ZnS_ value for CKL-3 (2.6 × 10^−8^ mol m^−2^ s^−1^) and CKL-4 (3.9 × 10^−9^ mol m^−2^ s^−1^) under oxic conditions at 20 °C were one or two orders of magnitude higher than the reference value (2.0 × 10^−10^ mol m^−2^ s^−1^). These findings indicate that the large *R*_Zn_ values for hydrothermal sulfides cannot be explained by only the simple oxidation reactions of sphalerite.

### Contribution of galvanic interactions to Zn and Pb dissolutions

One possible mechanism for sulfide mineral dissolution in seawater is galvanic interactions between different mineral couples [20, 25, 27, 28]. Sulfide minerals have semiconducting properties, and direct contact between sulfide minerals with different resting potentials may produce a galvanic effect [20, 29]. The minerals with the highest and lowest resting potentials act as cathodes and anodes, respectively. Cathodic minerals can be galvanically protected, while anodic minerals are easily dissolved through electronic interactions [28]. For example, the resting potentials of individual sulfide minerals in H_2_SO_4_ at pH 4 versus the standard hydrogen electrode were reported to be 0.66 V for pyrite, 0.63 V for marcasite, 0.46 V for sphalerite, and 0.40 V for galena [30]. These values show that sphalerite and galena in contact with iron disulfide minerals (i.e., pyrite and marcasite) can be anodically dissolved as follows (M = Zn, Pb):

3 $${\text{MS}} \to {\text{M}}^{ 2+ } + {\text{ S}}^{0} + {\text{ 2e}}^{ - }$$

Cathodic minerals (pyrite and marcasite) can be protected, and dissolved oxygen and/or oxidizing metal species such as ferric ions can be reduced by electrons on the cathodic mineral surface:

4 $${\text{O}}_{ 2} + {\text{ 4H}}^{ + } + {\text{ 4e}}^{ - } \to 2 {\text{H}}_{ 2} {\text{O}}$$

5 $${\text{Fe}}^{ 3+ } + {\text{ e}}^{ - } \to {\text{ Fe}}^{ 2+ }$$

**Fig. 7:**
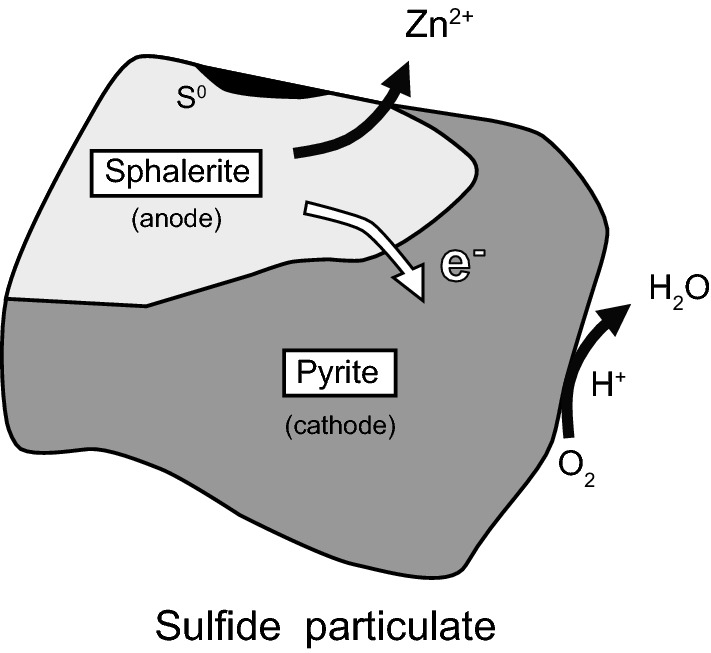
Schematic of the galvanic interaction between pyrite and sphalerite in hydrothermal sulfide particulate (modified from Liu et al. [27] and Fallon et al. [3])

The BSE image in Fig. 5d shows adjacent sphalerite (anode) and iron disulfides (cathode) in the sulfide particulates in CKL-4 (i.e., formation of galvanic couples), which released a large amount of Zn. In contrast, the release of Zn and Pb from the sulfide particulates of CKL-1 was restricted because they contained insufficient iron disulfide, even though there were large amounts of sphalerite and galena. The results of SEM observations imply that the galvanic couples of sphalerite and galena with iron disulfides may contribute to the release of Zn and Pb into seawater, as illustrated in Fig. 7.

The dissolution rates of Zn and Pb (i.e., *R*_ZnS_ and *R*_PbS_) for CKL-3 and CKL-4 were higher under oxic than anoxic conditions (Table 3). These results suggest that dissolved oxygen induced the galvanic dissolution of Zn and Pb from hydrothermal sulfides into seawater. Our findings are consistent with those of previous reports that showed galvanic interaction is suppressed in nitrogenated water because of the lower activity of dissolved oxygen [30]. As described above, Fe^3+^ can also act as an oxidant of the galvanic reaction; however, it cannot be present at high concentrations in seawater (only 0.2–0.3 nM) [18]. The Fe concentration greatly increased to 130 µM for CKL-3 under oxic conditions at 20 °C, but most filterable Fe (< 0.45 μm) was likely under the form of colloidal ferric oxyhydroxide at the pH of these experiments [31]. Thus, Fe^3+^ is unlikely to be involved in the galvanic interaction in this case. In our experiment, dissolved oxygen would mainly act as an electron acceptor of galvanic cells and promote Zn and Pb release from hydrothermal sulfide particulates (Fig. 7).

The sulfide particulates of CKL-2 contained significant amounts galvanic couples of pyrite with sphalerite. However, negative *R*_ZnS_ values were determined under oxic conditions (Table 3). As explained in the results section of the SEM observations, the particulates of CKL-2 were often coated with a silicate mineral (Fig. 5b). The coating by silicate minerals likely reduced the reactive surface area of sulfide particulates, which suppressed the release of metals from the particulates of CKL-2. As observed in CKL-1, precipitate formation and adsorption removal reactions of initially released metals in seawater became dominant rather than metal release, resulting in the dissolution rates of CKL-2 becoming negative.

In the case of CKL-3, the small pieces of sphalerite and galena in the pyrite particulates observed in the BSE image (Fig. 5c) may have been the origin of the large amounts of Zn and Pb released in seawater. Percentages of Zn (*D*_Zn_) and Pb (*D*_Pb_) for all samples dissolved in seawater relative to the initial sulfide sample under each experimental condition were shown in Table 4. These values were calculated from the Zn and Pb concentrations in the seawater after reaction for 18 h because the pH of the CKL-4 solution decreased greatly to 4.5 under oxic conditions at 20 °C during 18–30 h. The *D*_Zn_ and *D*_Pb_ values for CKL-3 were 2–3 times higher for Zn and almost 2 orders of magnitude for Pb higher than those for CKL-4 under oxic conditions at 20 °C (1.1% and 4.1%, respectively). The higher release of Zn and Pb from the small pieces of sphalerite and galena in CKL-3 is likely associated with the higher surface areas of the anodic and cathodic sulfide minerals [20, 28]. According to Kwong et al. [20], the current density generated by anodes of large surface area is low because the currents generated by the galvanic interactions are widely dispersed, and the low current density results in slow dissolution of the anodic sulfide minerals. As a result, small pieces of anodic sulfide minerals included in cathodic minerals dissolve more rapidly than large fragments. The high release of Zn from a pyrite-rich hydrothermal sulfide into artificial seawater has also been reported by Parry et al. [13] and Fuchida et al. [4]. Although they did not microscopically observe the fragments of sulfide particulates in their leaching experiments, Zn could be derived from small pieces of sphalerite and other Zn minerals in the pyrite particulates. Small inclusions of sulfide minerals would therefore be key factors in determining metal dissolution rates from hydrothermal sulfides through galvanic reactions, even if the amounts were too low to detect by XRD analysis.

**Table 4:** Percentages of Zn (*D*_Zn_) and Pb (*D*_Pb_) dissolved in seawater relative to the initial sulfide sample

			*D*_Zn_ (%)	*D*_Pb_ (%)			*D*_Zn_ (%)	*D*_Pb_ (%)
CKL-1	Anoxic	5 °C	0.0036	0.0053	Oxic	5 °C	0.0015	0.0038
		20 °C	0.0050	0.0077		20 °C	0.0035	0.0067
CKL-2	Anoxic	5 °C	0.12	0.026	Oxic	5 °C	0.047	0.015
		20 °C	0.15	0.049		20 °C	0.018	0.021
CKL-3	Anoxic	5 °C	0.11	0.98	Oxic	5 °C	0.35	1.2
		20 °C	0.19	2.3		20 °C	1.1	4.1
CKL-4	Anoxic	5 °C	0.11	0.063	Oxic	5 °C	0.25	0.079
		20 °C	0.14	0.17		20 °C	0.42	0.28

These values for all samples were calculated from the Zn and Pb concentrations in the seawater after reaction for 18 h because the pH of the CKL-4 solution decreased greatly to 4.5 under oxic conditions at 20 °C during 18–30 h and dissolution of Zn and Pb was greatly promoted under these conditions

For CKL-3 and CKL-4, the pH decreased gradually during the reaction, with the final pH for CKL-4 showing an especially great decrease to 4.5 under oxic conditions at 20 °C (Fig. 3). This decrease in pH may have been caused by the generation of acids (mainly H_2_SO_4_) during the oxidative dissolution of sulfide and/or dissolution of other sulfate minerals (e.g., CaSO_4_). The oxidation of elementary sulfur to thiosulfate also causes a pH decrease [32]. When galvanic interaction promotes the dissolution of sulfide minerals, elementary sulfur is accumulated on the anodic mineral surface, as shown in Fig. 7. Although we did not determine whether thiosulfate (S_2_O_3_^2−^) formed in seawater during the reaction, the oxidation of elementary sulfur formed by galvanic interaction might have contributed to the decreases in pH for CKL-3 and CKL-4.

Based on our experimental results and microscopic observations, Zn and Pb dissolution from hydrothermal sulfide minerals into seawater may have been promoted by galvanic interactions rather than simple oxidation of individual sulfide minerals. Although the dissolution rates of Zn and Pb are controlled by the physicochemical parameters of seawater, such as redox conditions and temperature, our results indicate that sulfide mineral couples and their micro configurations are important factors governing the galvanic dissolution rates. In particular, the presence of iron disulfide minerals with high resting potentials is needed for high metal release from natural hydrothermal sulfides in seawater.

### Metal release from hydrothermal sulfides in SMS-mining operations

The amounts of metals released from fresh hydrothermal sulfides (i.e., without long time exposure to the atmosphere) in our study are smaller than those from hydrothermal sulfide exposed to the atmosphere for a long time [4, 12, 13]. Furthermore, the metal release patterns for the fresh hydrothermal sulfides differed from those of hydrothermal sulfides exposed to the atmosphere for a long period of time. Specifically, the oxidized hydrothermal sulfides rapidly released various metals (Mn, Cu, Zn, As, Cd, Pb) into oxic seawater within several minutes [4, 12, 13], but the fresh hydrothermal sulfides used in this study primarily and gradually released Zn and Pb into seawater. These differences indicate that the initial oxidation states of the hydrothermal sulfide surfaces may result in different metal dissolution behaviors in seawater. Thus, the hydrothermal sulfides in each mining process would have different metal dissolution potentials.

In general, natural hydrothermal sulfides beneath the seafloor exist in a less-oxidized state because they are often covered by insoluble oxides and/or sulfates [3, 33]. Under such conditions, high rates of metal dissolution are suppressed. However, our results indicate that a small amount of Zn and Pb might be released from hydrothermal sulfide when a fresh sulfide surface is exposed to seawater, even though the amounts released are much lower than when hydrothermal sulfide is exposed to the atmosphere.

According to the SMS-mining model proposed by several contractors, hydrothermal minerals are crushed using a seafloor mining tool, lifted from the seafloor to a mining support vessel through a riser pipe, and dewatered onboard [34, 35]. This process increases the opportunities for hydrothermal sulfides with large interface areas to make contact with seawater. The metal released from hydrothermal sulfide is estimated to be limited in the open marine environment because of the alkalescence and high-buffering capacity of seawater [3, 5]; however, metal rich seawater might be generated in the lifting process because large amounts of hydrothermal sulfide mineral slurry flow with seawater in the riser pipe. Moreover, metal release from crushed hydrothermal sulfides is likely to be greatly accelerated under warm oxic conditions, such as the surface environment, than under cold anoxic conditions such as those found on the seafloor. The dewatering process on the vessel would also discharge metal rich seawater. Therefore, adequate monitoring of seawater quality and onboard treatment of the discharge materials may be required to minimize the negative impacts of SMS-mining on marine environments.

## Conclusion

Our experiments clearly demonstrated that metal dissolution from hydrothermal sulfides into seawater may occur without long term exposure to the atmosphere. Metal dissolution rates depended on the sulfide mineral assemblage and their surface area; therefore, the presence of high-rest-potential iron disulfide minerals (i.e., pyrite and marcasite) may be a primary factor in inducing dissolution of low-rest-potential minerals (i.e., sphalerite and galena). These results support the dissolution mechanisms from hydrothermal sulfides discussed in a previous study [3]. The dissolution rates of Zn and Pb also depended on physicochemical parameters and were enhanced under higher redox and temperature conditions. These findings imply that metals dissolution could be accelerated when hydrothermal sulfides are lifted to the surface, as surface conditions are more oxic and warmer than seafloor conditions.

Metal-contaminated drainage generation and the toxic effects of metal contaminants on marine ecosystems are important issues that need to be considered when mitigating the impacts of SMS-mining on marine environments. The results of our onboard leaching experiments investigating fresh hydrothermal sulfides will help achieve realistic evaluations of drainage generation during SMS-mining operations.

## Additional file

**Additional file 1: Table S1.** pH and concentrations of Fe, Cu, Zn and Pb in seawater from powdered core samples at different temperature and redox conditions. **Figure S1.** Photographs of hydrothermal mineral cores for onboard leaching experiment: (a) C9026A 7X-CC (CKL-1), (b) C9027B 1X-CC (CKL-2), (c) C9028A 7S-CC (CKL-3), and (d) C9028A 1H-7 (CKL-4). **Figure S2.** Images of onboard leaching experiment: (a) an operation in the anaerobic chamber and (b) sample reactions in the water baths. **Figure S3.** Changes in *E*_h_ (V, SHE) for (a) CKL-1, (b) CKL-2, (c) CKL-3, and (d) CKL-4 solutions under different redox and temperature conditions. Plots show mean values of duplicates, and error bars indicate range of duplicate (difference between the max and min values).

## Abbreviations

SMS: seafloor massive sulfide
BET: Brunauer–Emmett–Teller
ORP: oxidation–reduction potential
PTFE: poly(tetrafluoroethylene)
XRD: X-ray diffraction
SEM: scanning electron microscopy
EDS: energy-dispersive X-ray spectroscopy
BSE: backscattered electron
SHE: standard hydrogen electrode
SE: secondary electron

## References

[1] Hageman PL, Seal RR, Diehl SF, Piatak NM, Lowers HA (2015). Evaluation of selected static methods used to estimate element mobility, acid-generating and acid-neutralizing potentials associated with geologically diverse mining wastes. Appl Geochem.

[2] Simpson SL, Spadaro DA (2016). Bioavailability and chronic toxicity of metal sulfide minerals to benthic marine invertebrates: implications for deep sea exploration, mining and tailings disposal. Environ Sci Technol.

[3] Fallon EK, Petersen S, Brooker RA, Scott TB (2017). Oxidative dissolution of hydrothermal mixed-sulphide ore: an assessment of current knowledge in relation to seafloor massive sulphide mining. Ore Geol Rev.

[4] Fuchida S, Yokoyama A, Fukuchi R, Ishibashi J, Kawagucci S, Kawachi M, Koshikawa H (2017). Leaching of metals and metalloids from hydrothermal ore particulates and their effects on marine phytoplankton. ACS Omega..

[5] Bilenker LD, Romano GY, McKibben MA (2016). Kinetics of sulfide mineral oxidation in seawater: implications for acid generation during in situ mining of seafloor hydrothermal vent deposits. Appl Geochem.

[6] Williamson MA, Rimstidt JD (1994). The kinetics and electrochemical rate-determining step of aqueous pyrite oxidation. Geochim Cosmochim Acta.

[7] Holmes PR, Crundwell FK (2000). The kinetics of the oxidation of pyrite by ferric ions and dissolved oxygen: an electrochemical study. Geochim Cosmochim Acta.

[8] Weisener CG, Smart RSC, Gerson AR (2004). A comparison of the kinetics and mechanism of acid leaching of sphalerite containing low and high concentrations of iron. Int J Miner Proc.

[9] Acero P, Cama J, Ayora C (2007). Sphalerite dissolution kinetics in acidic environment. Appl Geochem.

[10] Heidel C, Tichomirowa M, Breitkopf C (2011). Sphalerite oxidation pathways detected by oxygen and sulfur isotope studies. Appl Geochem.

[11] Heidel C, Tichomirowa M (2011). Galena oxidation investigations on oxygen and sulphur isotopes. Isotopes Environ Health Stud..

[12] Simpson SL, Angel B, Hamilton I, Spadaro DA, Binet M (2007) Water and sediment characterization and toxicity assessment for the Solwara 1 Project. CSIRO Land and Water Science Report. Coffey Natural Systems Pty Ltd

[13] Parry DL (2008) Solwara 1 Project elutriate Report phase 1 and 2

[14] US Environmental Protection Agency. National recommended water quality criteria for—aquatic life criteria table. https://www.epa.gov/wqc/national-recommended-water-quality-criteria-aquatic-life-criteria-table. Accessed 1 Oct 2018

[15] Sohrin Y, Urushihara S, Nakatsuka S, Kono T, Higo E, Minami T, Norisuye K, Umetani S (2008). Multielemental determination of GEOTRACES key trace metals in seawater by ICPMS after preconcentration using an ethylenediaminetriacetic acid chelating resin. Anal Chem.

[16] Morse JW (1991). Oxidation kinetics of sedimentary pyrite in seawater. Geochim Cosmochim Acta.

[17] Cook NJ, Ciobanu CL, Pring A, Skinner W, Shimizu M, Danyushevsky L, Saini-Eidukat B, Melcher F (2009). Trace and minor elements in sphalerite: a LA-ICPMS study. Geochim Cosmochim Acta.

[18] Liu X, Millero FJ (1999). The solubility of iron in sodium chloride solution. Geochim Cosmochim Acta.

[19] Angel BM, Apte SC, Batley GE, Raven MD (2016). Lead solubility in seawater: an experimental study. Environ Chem.

[20] Kwong YTJ, Swerhone GW, Lawrence JR (2013). Galvanic sulphide oxidation as a metal-leaching mechanism and its environmental implications. Geochem Explor Environ Anal.

[21] Gleisner M, Herbert RB, Kockum PCF (2006). Pyrite oxidation by *Acidithiobacillus ferrooxidans* at various concentrations of dissolved oxygen. Chem Geol.

[22] Tichomirowa M, Junghans M (2009). Oxygen isotope evidence for sorption of molecular oxygen to pyrite surface sites and incorporation into sulfate in oxidation experiments. Appl Geochem.

[23] Johnson CA (1986). The regulation of trace element concentrations in river and estuarine waters contaminated with acid mine drainage: the adsorption of Cu and Zn on amorphous Fe oxyhydroxides. Geochim Cosmochim Acta.

[24] Heidel C, Tichomirowa M, Junghans M (2013). Oxygen and sulfur isotope investigations of the oxidation of sulfide mixtures containing pyrite, galena, and sphalerite. Chem Geol.

[25] Tsang JJ, Parry DL (2004). Metal mobilization from complex sulfide ore concentrate: effect of light and pH. Aust J Chem.

[26] Chandra AP, Gerson AR (2010). The mechanisms of pyrite oxidation and leaching: a fundamental perspective. Surf Sci Rep.

[27] Liu Q, Li H, Zhou L (2008). Galvanic interactions between metal sulfide minerals in a flowing system: implications for mines environmental restoration. Appl Geochem.

[28] Chopard A, Plante B, Benzaazoua M, Bouzahzah H, Marion P (2017). Geochemical investigation of the galvanic effects during oxidation of pyrite and base-metals sulfides. Chemosphere.

[29] Cruz R, Luna-Sánchez RM, Lapidus GT, González I, Monroy M (2005). An experimental strategy to determine galvanic interactions affecting the reactivity of sulfide mineral concentrates. Hydrometallurgy.

[30] Rao SR, Finch JA (1988). Galvanic interaction studies on sulphide. Can Met Quart.

[31] Lewin J, Chen C (1973). Changes in the concentration of soluble and particulate iron in seawater enclosed in containers. Limnol Oceanogr.

[32] Maki Y (1987). Effect of dissolved oxygen concentration on the biological oxidation of sulfide and elemental sulfur by the A-type sulfurturf growing in hot spring effluents. J Gen Appl Microbiol.

[33] Feely RA, Lewison M, Massoth GJ, Robert-Baldo G, Lavelle JW, Byrne RH, Von Damm KL, Curl HC (1987). Composition and dissolution of black smoker particulates from active vents on the Juan-De-Fuca ridge. J Geophys Res Solid Earth.

[34] Collins PC, Croot P, Carlsson J, Colaço A, Grehan A, Hyeong K, Kennedy R, Mohn C, Smith S, Yamamoto H, Rowden A (2013). A primer for the environmental impact assessment of mining at seafloor massive sulfide deposits. Mar Pol.

[35] Narita T, Oshika J, Okamoto N, Toyohara T, Miwa T (2015). Summary of environmental impact assessment for mining seafloor massive sulfides in Japan. J Ship Ocean Eng.

