# Supplementary material for: Onboard experiment investigating metal leaching of fresh hydrothermal sulfide cores into seawater
Source: Geochem Trans. 2018 Dec 6;19:15. doi: 10.1186/s12932-018-0060-9 (PMC6755555; doi:10.1186/s12932-018-0060-9)
Supplement: Supplementary file 1 — Additional file 1: Table S1. pH and concentrations of Fe, Cu, Zn and Pb in seawater from powdered core samples at different temperature and redox conditions. Figure S1. Photographs of hydrothermal mineral cores for onboard leaching experiment: (a) C9026A 7X-CC (CKL-1), (b) C9027B 1X-CC (CKL-2), (c) C9028A 7S-CC (CKL-3), and (d) C9028A 1H-7 (CKL-4). Figure S2. Images of onboard leaching experiment: (a) an operation in the anaerobic chamber and (b) sample reactions in the water baths. Figure S3. Changes in Eh (V, SHE) for (a) CKL-1, (b) CKL-2, (c) CKL-3, and (d) CKL-4 solutions under different redox and temperature conditions. Plots show mean values of duplicates, and error bars indicate range of duplicate (difference between the max and min values). [file 12932_2018_60_MOESM1_ESM.docx]

**Additional Information**

**Onboard experiment investigating metal leaching of fresh hydrothermal sulfide cores into seawater**

Shigeshi Fuchida^1,*^, Jun-ichiro Ishibashi^2^, Kazuhiko Shimada^2^, Tatsuo Nozaki^3,4,5,6^, Hideaki Kumagai^3^, Masanobu Kawachi^7^, Yoshitaka Matsushita^8^, Hiroshi Koshikawa^1^

^1^Marine Environment Section, Center for Regional Environmental Research, National Institute for Environmental Studies (NIES), 16-2 Onogawa, Tsukuba, Ibaragi 305-8506, Japan

^2^Department of Earth and Planetary Sciences, Faculty of Science, Kyushu University, 744 Motooka, Nishi-ku, Fukuoka 819-0395, Japan

^3^Research and Development (R&D) Center for Submarine Resources, Japan Agency for Marine-Earth Science and Technology (JAMSTEC), 2-15 Natsushima-cho, Yokosuka, Kanagawa 237-0061, Japan

^4^Frontier Research Center for Energy and Resources, The University of Tokyo, 7-3-1 Hongo, Bunkyo-ku, Tokyo 113-8656, Japan

^5^Department of Planetology, Kobe University, 1-1 Rokkodai-cho, Nada-ku, Kobe, Hyogo 657-8501, Japan

^6^Ocean Resources Research Center for Next Generation, Chiba Institute of Technology, 2-17-1 Tsudanuma, Narashino, Chiba 275-0016, Japan

^7^Biodiversity Resource Conservation Office, Center for Environmental Biology and Ecosystem Studies, National Institute for Environmental Studies (NIES), 16-2 Onogawa, Tsukuba, Ibaragi 305-8506, Japan

^8^Research Network and Facility Services Division, the National Institute for Materials Science (NIMS), 1-2-1 Sengen, Tsukuba, Ibaragi 305-0047, Japan

| **CKL-1 (C9026A 7X-CC)**  C9026A | | |  |  |  |  |  |  |  |  |  |  |  |  |
| --- | --- | --- | --- | --- | --- | --- | --- | --- | --- | --- | --- | --- | --- | --- |
| (oxic) | Time (h) | pH | Fe (μM) | Cu (μM) | Zn (μM) | Pb (μM) |  | (anoxic) | Time (h) | pH | Fe (μM) | Cu (μM) | Zn (μM) | Pb (μM) |
| 5°C | 1 | 8.0 | ^*^- | - | 17 | 3.2 |  | 5°C | 1 | 8.0 | - | - | 8.1 | 3.7 |
|  | 4 | 8.2 | - | - | 17 | 1.8 |  |  | 4 | 8.1 | - | - | 12 | 2.2 |
|  | 10 | 8.6 | - | - | 7.4 | 1.3 |  |  | 10 | 8.2 | - | - | 9.3 | 1.2 |
|  | 18 | 8.9 | - | - | 1.8 | 0.93 |  |  | 18 | 8.6 | - | - | 4.2 | 1.3 |
|  | 30 | 9.0 | - | - | 1.1 | 0.74 |  |  | 30 | 8.9 | - | - | 1.8 | 1.1 |
|  |  |  |  |  |  |  |  |  |  |  |  |  |  |  |
| 20°C | 1 | 8.0 | - | - | 15 | 3.7 |  | 20°C | 1 | 8.0 | - | - | 5.8 | 3.1 |
|  | 4 | 8.3 | - | - | 11 | 2.3 |  |  | 4 | 8.0 | - | - | 9.3 | 3.0 |
|  | 10 | 8.5 | - | - | 6.3 | 1.8 |  |  | 10 | 8.2 | - | - | 8.2 | 2.0 |
|  | 18 | 8.7 | - | - | 4.0 | 1.6 |  |  | 18 | 8.3 | - | - | 5.8 | 1.9 |
|  | 30 | 8.8 | - | - | 2.1 | 1.5 |  |  | 30 | 8.7 | - | - | 1.8 | 1.5 |
|  |  |  |  |  |  |  |  |  |  |  |  |  |  |  |
|  |  |  |  |  |  |  |  |  |  |  |  |  |  |  |
| **CKL-2 (C9027B 1X-CC)** | | |  |  |  |  |  |  |  |  |  |  |  |  |
| (oxic) | Time (h) | pH | Fe (μM) | Cu (μM) | Zn (μM) | Pb (μM) |  | (anoxic) | Time (h) | pH | Fe (μM) | Cu (μM) | Zn (μM) | Pb (μM) |
| 5°C | 1 | 7.7 | - | - | 38 | 6.0 |  | 5°C | 1 | 7.7 | - | - | 25 | 1.9 |
|  | 4 | 7.7 | - | - | 54 | 3.9 |  |  | 4 | 7.7 | - | - | 26 | 2.0 |
|  | 10 | 8.1 | - | - | 50 | 1.8 |  |  | 10 | 7.8 | - | - | 40 | 3.1 |
|  | 18 | 8.4 | - | - | 20 | 1.4 |  |  | 18 | 7.7 | - | - | 50 | 2.4 |
|  | 30 | 8.6 | - | - | 5.0 | 1.3 |  |  | 30 | 7.8 | - | - | 52 | 1.8 |
|  |  |  |  |  |  |  |  |  |  |  |  |  |  |  |
| 20°C | 1 | 7.6 | - | - | 47 | 10 |  | 20°C | 1 | 7.7 | - | - | 7.9 | 0.76 |
|  | 4 | 7.6 | - | - | 57 | 5.1 |  |  | 4 | 7.7 | - | - | 13 | 1.3 |
|  | 10 | 8.3 | - | - | 17 | 2.3 |  |  | 10 | 7.7 | - | - | 43 | 4.1 |
|  | 18 | 8.6 | - | - | 7.8 | 2.0 |  |  | 18 | 7.6 | - | - | 63 | 4.5 |
|  | 30 | 8.2 | - | - | 18 | 3.0 |  |  | 30 | 7.8 | - | - | 54 | 4.1 |

**Table S1**. pH and concentrations of Fe, Cu, Zn and Pb in seawater from powdered core samples at different temperature and redox conditions.

**Table S1.** (continued)

| **CKL-3 (C9028A 7S-CC)** | | |  |  |  |  |  |  |  |  |  |  |  |  |
| --- | --- | --- | --- | --- | --- | --- | --- | --- | --- | --- | --- | --- | --- | --- |
| (oxic) | Time (h) | pH | Fe (μM) | Cu (μM) | Zn (μM) | Pb (μM) |  | (anoxic) | Time (h) | pH | Fe (μM) | Cu (μM) | Zn (μM) | Pb (μM) |
| 5°C | 1 | 7.5 | - | - | 8.7 | 3.5 |  | 5°C | 1 | 7.8 | - | - | 2.7 | 1.2 |
|  | 4 | 7.5 | - | - | 13 | 3.6 |  |  | 4 | 7.7 | - | - | 4.4 | 1.8 |
|  | 10 | 7.3 | - | - | 21 | 3.7 |  |  | 10 | 7.7 | - | - | 7.2 | 3.3 |
|  | 18 | 7.2 | - | - | 32 | 3.7 |  |  | 18 | 7.6 | - | - | 10 | 2.9 |
|  | 30 | 6.7 | - | - | 47 | 3.8 |  |  | 30 | 7.4 | - | - | 15 | 3.4 |
|  |  |  |  |  |  |  |  |  |  |  |  |  |  |  |
| 20°C | 1 | 7.3 | - | - | 8.2 | 4.2 |  | 20°C | 1 | 7.9 | - | - | 1.0 | 0.55 |
|  | 4 | 7.3 | - | - | 19 | 4.3 |  |  | 4 | 7.5 | - | - | 6.9 | 4.5 |
|  | 10 | 7.1 | - | 0.38 | 44 | 4.8 |  |  | 10 | 7.5 | - | - | 13 | 6.6 |
|  | 18 | 6.5 | - | 0.91 | 96 | 12 |  |  | 18 | 7.4 | - | - | 17 | 6.7 |
|  | 30 | 4.5 | 130 | 23 | 190 | 130 |  |  | 30 | 7.2 | - | - | 21 | 5.9 |
|  |  |  |  |  |  |  |  |  |  |  |  |  |  |  |
| **CKL-4 (C9028A 1H-7)** | | |  |  |  |  |  |  |  |  |  |  |  |  |
| (oxic) | Time (h) | pH | Fe (μM) | Cu (μM) | Zn (μM) | Pb (μM) |  | (anoxic) | Time (h) | pH | Fe (μM) | Cu (μM) | Zn (μM) | Pb (μM) |
| 5°C | 1 | 7.4 | - | - | 93 | 9.7 |  | 5°C | 1 | 7.5 | - | - | 62 | 17 |
|  | 4 | 7.5 | - | - | 110 | 6.3 |  |  | 4 | 7.6 | - | - | 67 | 7.3 |
|  | 10 | 7.5 | - | - | 130 | 2.7 |  |  | 10 | 7.6 | - | - | 76 | 3.7 |
|  | 18 | 7.3 | - | - | 200 | 3.3 |  |  | 18 | 7.5 | - | - | 90 | 2.6 |
|  | 30 | 7.2 | - | - | 310 | 5.2 |  |  | 30 | 7.4 | - | - | 120 | 3.2 |
|  |  |  |  |  |  |  |  |  |  |  |  |  |  |  |
| 20°C | 1 | 7.3 | - | - | 100 | 14 |  | 20°C | 1 | 7.5 | - | - | 69 | 14 |
|  | 4 | 7.4 | - | - | 130 | 12 |  |  | 4 | 7.6 | - | - | 80 | 7.5 |
|  | 10 | 7.3 | - | - | 190 | 6.6 |  |  | 10 | 7.4 | - | - | 85 | 6.4 |
|  | 18 | 7.0 | - | - | 340 | 12 |  |  | 18 | 7.4 | - | - | 110 | 7.1 |
|  | 30 | 6.9 | - | - | 610 | 21 |  |  | 30 | 7.1 | - | - | 140 | 11 |

*-: below limit of quantification


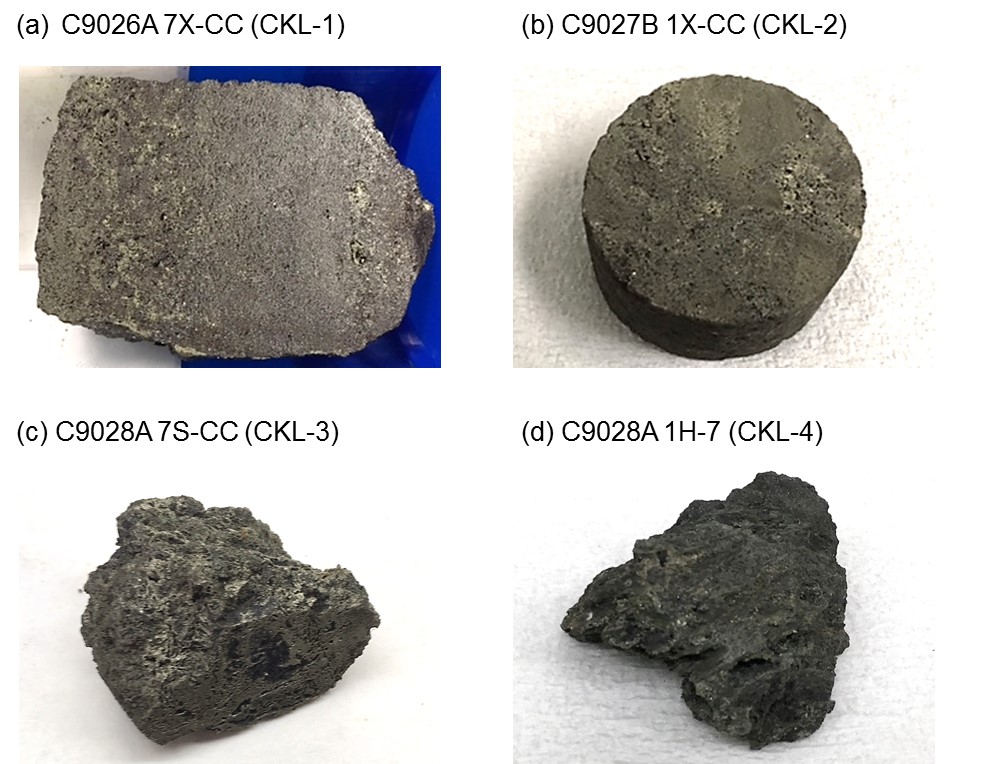


**Figure S1** Photographs of hydrothermal mineral cores for onboard leaching experiment: (a) C9026A 7X-CC (CKL-1), (b) C9027B 1X-CC (CKL-2), (c) C9028A 7S-CC (CKL-3), and (d) C9028A 1H-7 (CKL-4).

(a)

(b)


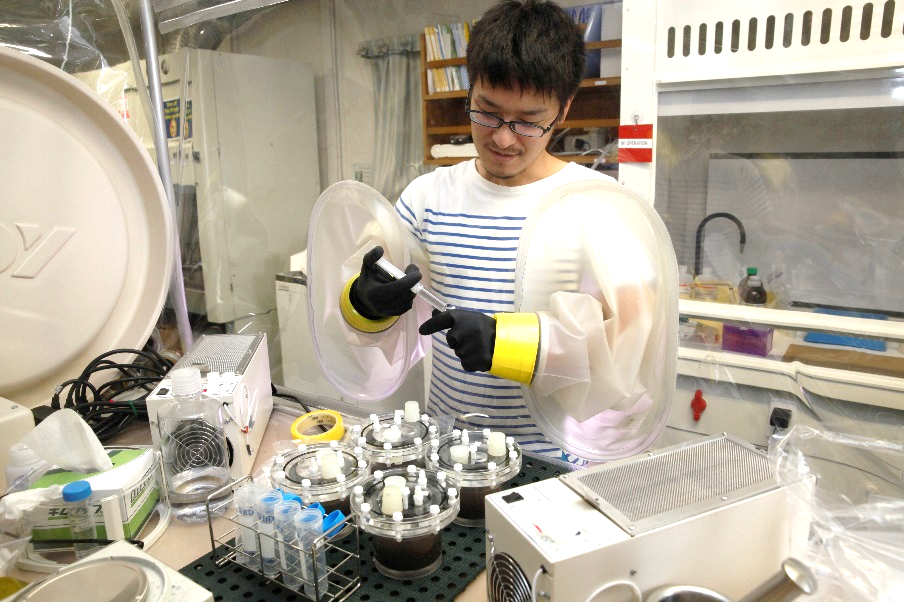

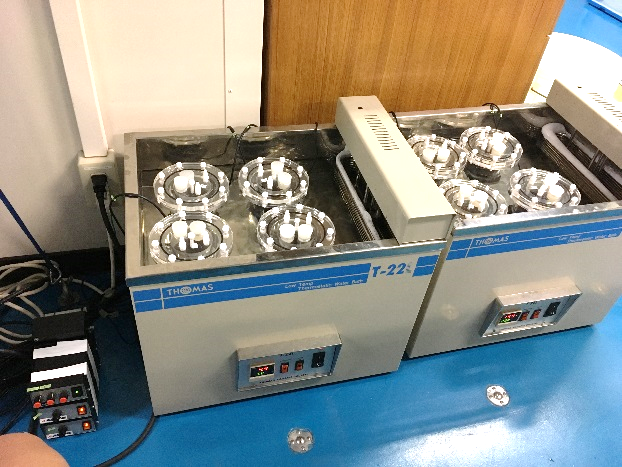


**Figure S2**. Images of onboard leaching experiment: (a) an operation in the anaerobic chamber and (b) sample reactions in the water baths.


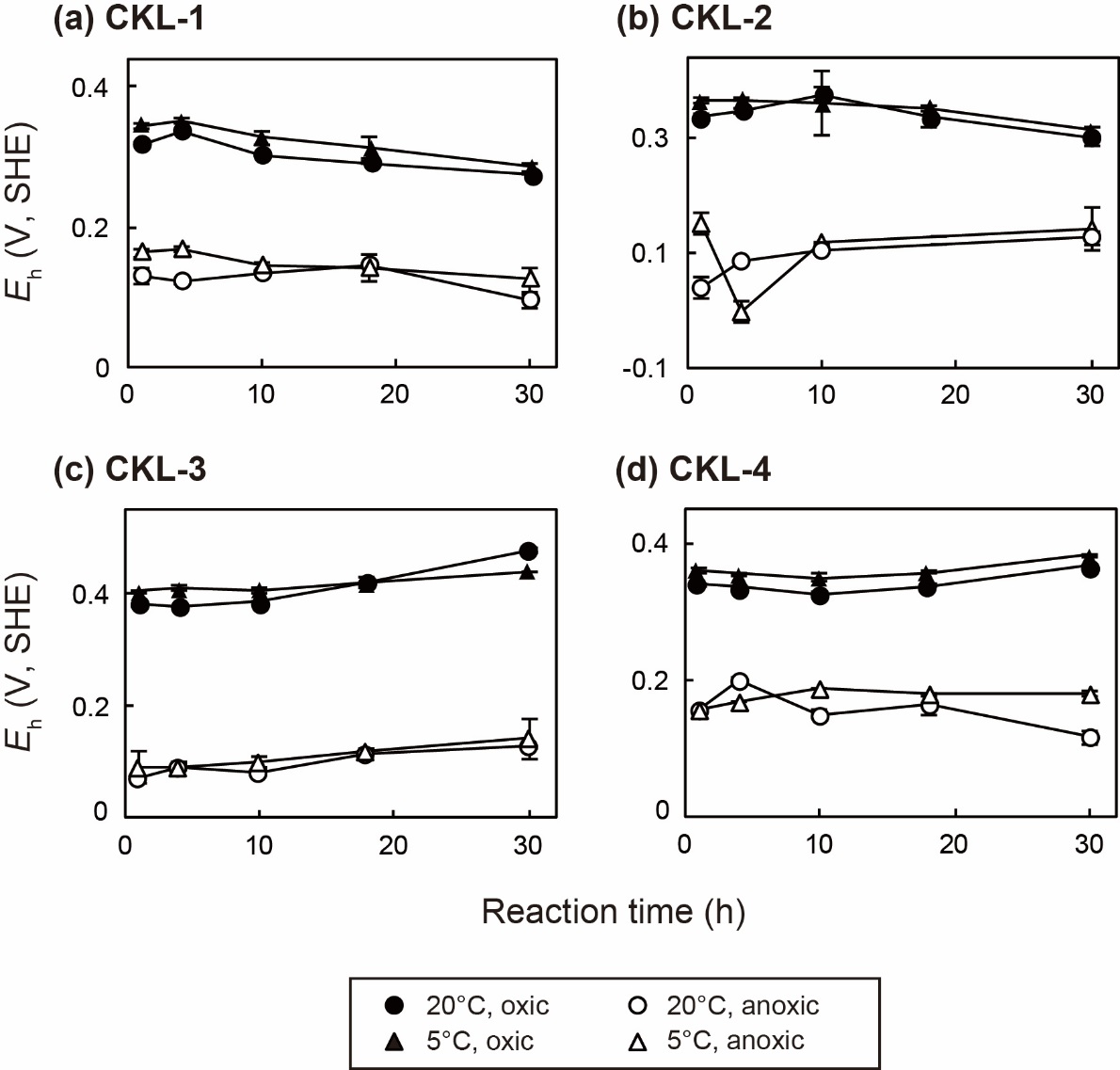


**Figure S3**. Changes in *E*_h_ (V, SHE) for (a) CKL-1, (b) CKL-2, (c) CKL-3, and (d) CKL-4 solutions under different redox and temperature conditions. Plots show mean values of duplicates, and error bars indicate range of duplicate (difference between the max and min values).
